# Causal Uncertainty-Decomposed Ensemble Learning for Confounding-Aware Skin Cancer Detection in Dermoscopic Images

**DOI:** 10.3390/diagnostics16152453

**Published:** 2026-08-03

**Authors:** Mohd. Faheem Khan, Khurshid Ahmad

**Affiliations:** 1 Department of Biotechnology and Microbiology, Khandelwal College of Management, Science and Technology, M.J.P. Rohilkhand University, Bareilly 243122, Uttar Pradesh, India; 2 Department of Health Informatics, College of Applied Medical Sciences, Qassim University, Buraydah 51452, Saudi Arabia

**Keywords:** skin cancer detection, dermoscopy, causal representation learning, confounding, uncertainty quantification, Bayesian neural networks, ensemble learning, ISIC 2019, calibration, selective classification

## Abstract

**Background/Objectives:** Automated dermoscopic image analysis can assist research on early skin cancer detection, but deep learning models may learn acquisition-related shortcuts, including terminal hairs, illumination gradients, gel bubbles, shadows and measurement markings. This study evaluates a causal uncertainty-decomposed ensemble (CUDE) for confounding-aware skin-lesion classification within the ISIC 2019 benchmark setting. **Methods:** CUDE uses a structured variational autoencoder (SVAE) to impose separate lesion-relevant and acquisition-related nuisance latent heads. Three stochastic latent-space experts are trained on complementary streams and are integrated by a Dirichlet-based fusion module trained with nuisance sampling. The causal graph is used as a modeling prior rather than proof of causal identification. **Results:** Using a stratified internal split of the labeled ISIC 2019 training collection, CUDE achieved a balanced accuracy of 89.7% and an AUROC of 0.972. Under the predefined synthetic artifact protocol, CUDE showed a relative performance drop of 5.0%, compared with 9.6% for the deep ensemble. Deferring the 10% most uncertain cases increased non-deferred accuracy from 89.7% to 93.2% and reduced false-negative rates for melanoma, BCC and SCC in the non-deferred subset. **Conclusions:** Within the evaluated ISIC 2019 split and controlled synthetic corruption protocol, structured latent separation, stochastic expert diversity and Dirichlet fusion were associated with improved benchmark robustness and calibration. These results should not be interpreted as evidence of broad real-world robustness or clinical deployment readiness; external multi-center, device-diverse and skin-tone-diverse validation remains required.

## 1. Introduction

Skin cancer is among the most common malignancies worldwide [1]. Early detection remains central to improving outcomes in skin neoplasms [2]. Automated dermoscopic image analysis has advanced substantially with deep convolutional and transformer-based architectures, including ResNet and Vision Transformer models [3,4]. However, strong benchmark performance does not by itself establish clinical readiness. Dermoscopic AI systems must be evaluated carefully for dataset shift, imaging artifacts and population-level generalizability before they can be considered for clinical use [5,6].

A major limitation of many image-classification models is their tendency to learn associations that are predictive within the training distribution but are not causally related to disease [7]. Dermoscopic images may contain terminal hairs, measurement markings, illumination gradients, gel bubbles, shadows and device-specific color profiles [8,9,10]. These nuisance variables can become spuriously associated with diagnostic labels, causing a model to attend to acquisition artifacts rather than lesion morphology [9,11]. When these correlations change across datasets, apparent benchmark performance may not translate into stable performance on images acquired under different protocols [6].

Ensemble learning is frequently used to improve robustness by aggregating predictions from multiple classifiers [12]. In skin-lesion classification, recent studies have combined convolutional backbones, transformer models and voting or stacking strategies to improve accuracy [13,14]. However, most ensembles operate on raw pixels or shared feature spaces and therefore do not explicitly separate etiological lesion characteristics from acquisition-related nuisance variation. As a result, multiple base learners may still exploit similar artifact-driven shortcuts.

Causal inference provides a principled framework for addressing confounding. Structural causal models can represent the relationships among lesion attributes, nuisance artifacts and diagnostic labels, while backdoor adjustment can reduce confounding by conditioning on, intervening on or marginalizing nuisance variables [15,16,17]. In parallel, probabilistic deep learning provides uncertainty estimates that are important for clinical decision support, particularly when models encounter ambiguous or out-of-distribution cases [18,19]. Combining causal representation learning with uncertainty-aware prediction may therefore improve both diagnostic robustness and interpretability.

This study proposes CUDE, a causal-prior and uncertainty-aware ensemble for skin-lesion classification in dermoscopic images. The framework imposes a structured separation between lesion-relevant and nuisance latent representations, assigns the resulting streams to specialized stochastic experts and fuses their predictions through a Dirichlet-based meta-learner trained with nuisance sampling. The objective is to examine whether this design improves classification performance, calibration, selective deferral and resistance to a predefined synthetic artifact protocol using the ISIC 2019 benchmark dataset. The study does not claim that the imposed causal graph has been empirically identified, nor does it claim broad real-world robustness or clinical deployment readiness without external validation.

## 2. Related Work

This study intersects four research areas: causal disentanglement in medical imaging, ensemble learning for skin-lesion classification, uncertainty quantification in deep learning and confounding-aware model evaluation. Existing methods have improved diagnostic accuracy, but fewer approaches jointly address artifact-driven confounding, representation-level expert diversity and calibrated clinical uncertainty.

### 2.1. Causal Disentanglement in Medical Imaging

Causal representation learning aims to separate etiologically meaningful factors from variables that are associated with the data-generation process but are not causally responsible for the outcome. Variational autoencoders and structured variants have been used to obtain latent representations that are more interpretable and controllable [20,21]. In medical imaging, this distinction is particularly important because artifacts, scanner-specific characteristics and acquisition protocols may be strongly associated with patient groups or diagnostic labels without being biologically causal [17,22].

For skin cancer diagnosis, causal disentanglement is relevant because dermoscopic images frequently contain non-lesion cues. A model that learns hair shafts, ruler marks or illumination patterns as diagnostic features may perform well on a benchmark but fail on images acquired using different protocols. The present framework therefore imposes a structured separation between lesion morphology and nuisance artifacts before classification.

### 2.2. Ensemble Methods for Skin-Lesion Classification

Ensemble learning improves predictive stability by combining multiple learners [12]. Prior studies in dermoscopic image classification have used majority voting, stacking, deep ensembles and combinations of pretrained backbones such as EfficientNet, DenseNet, ResNet and transformer-based models [13,14]. These approaches often achieve higher accuracy than individual models, particularly in imbalanced multi-class classification settings.

Standard ensembles generally assume that model diversity is sufficient to reduce error. If all ensemble members are trained on the same confounded pixel distribution, however, they may converge on similar artifact-based shortcuts. CUDE differs by enforcing diversity at the representation level: one expert operates on causal lesion features, one models nuisance interactions and one captures cross-covariate patterns. This design allows the fusion module to assign greater weight to robust causal evidence when nuisance corruption is high.

### 2.3. Uncertainty Quantification in Deep Learning

Reliable uncertainty estimation is important for selective classification and risk-aware model evaluation. Aleatoric uncertainty reflects irreducible ambiguity in the data, whereas epistemic uncertainty reflects uncertainty caused by limited knowledge, finite training data or distributional shift [18,19]. Bayesian neural networks and Monte Carlo dropout provide practical approximations for stochastic prediction in deep models [23,24]. Evidential approaches further model uncertainty by predicting parameters of a distribution over class probabilities [25].

In CUDE, each expert produces stochastic predictions through Monte Carlo dropout. The fusion module transforms expert-level mean probabilities and stochastic variances into Dirichlet concentration parameters, yielding a predictive probability vector and a total predictive uncertainty score. Accordingly, Monte Carlo dropout is treated as a proxy for epistemic variability, whereas Dirichlet concentration summarizes total predictive uncertainty; these outputs are not interpreted as clinically validated, separable aleatoric and epistemic mechanisms.

### 2.4. Novelty of the Present Study

Prior approaches have addressed individual components of the problem, including skin-lesion classification, ensemble learning, uncertainty estimation and causal representation [26,27]. Recent VAE work also highlights that latent regularization and KL-loss design influence representation quality and computational complexity [28]. The novelty of CUDE lies in testing a combined confounding-aware architecture in which latent separation, stochastic expert diversity and Dirichlet fusion are evaluated together on ISIC 2019. The evidence generated in this study is limited to the ISIC 2019 benchmark dataset and the predefined synthetic artifacts described in the following section.

## 3. Materials and Methods

The CUDE framework comprises three linked stages: structured latent representation learning, stochastic expert prediction and confounding-aware fusion. This section describes the causal assumptions, architecture, implementation configuration, dataset split, nuisance-reference construction, GNN specification, latent-separation probe analysis, evaluation metrics, robustness protocol, deferral thresholds and computational considerations used in the study. Additional configuration files and split-manifest instructions are provided in the public repository.

### 3.1. Causal Preliminaries and Uncertainty Decomposition

The dermoscopic image-generation process was represented using a structural causal model. Let X denote the observed dermoscopic image, Zc the latent lesion-relevant characteristics, Zn the acquisition-related nuisance variables and Y the disease label. The central modeling assumption is that Y is primarily determined by lesion-relevant information, whereas X is generated from both lesion characteristics and acquisition-related nuisance factors. This graph is used once as an imposed structural prior to guide representation learning; it is not presented as empirical proof that nuisance factors are independent of disease in the collected data.

Zc → Y, (Zc, Zn) → X, Zn ↛ Y(1)

The objective is to estimate predictions that depend primarily on lesion-relevant features while reducing the influence of nuisance artifacts. Conceptually, the desired prediction can be expressed as the backdoor-adjusted expectation in Equation (2). During training, the fusion module samples nuisance configurations from a fixed reference distribution to discourage model decisions from relying on accidental artifact-label correlations. This procedure approximates confounding control within the benchmark setting; it does not establish formal causal identification under untested assumptions.

(2) p(Y | do(Zc))=∫ p(Y | Zc, zn) p(zn) dzn

The framework reports stochastic predictive uncertainty in two complementary forms. Monte Carlo dropout across repeated forward passes provides an epistemic-variability proxy, whereas the final Dirichlet strength provides a scalar total uncertainty score. We therefore avoid interpreting the benchmark results as a clinically validated separation of aleatoric and epistemic uncertainty. The uncertainty outputs are used for calibration assessment and exploratory selective-classification analysis.

### 3.2. Causal Disentanglement and Confounding-Aware Ensemble Architecture

CUDE consists of an SVAE for structured latent separation, three stochastic experts for subspace-specific prediction and a Dirichlet-based fusion module with nuisance sampling. The design encourages, but does not guarantee, separation between lesion morphology and acquisition artifacts and aggregates expert predictions according to predictive evidence and uncertainty. The final configuration used zc = 128, zn = 64, dropout = 0.20, 30 Monte Carlo forward passes at inference and seed = 42 for data splitting and model initialization where applicable.

### 3.3. Causal Disentanglement via Structured Variational Autoencoder

The SVAE maps each 256 × 256 dermoscopic image into two latent subspaces: zc for lesion-relevant information and zn for acquisition-related nuisance variation. The encoder contains four convolutional blocks with batch normalization and ReLU activations, followed by separate mean and log-variance heads for zc and zn. The decoder reconstructs the image from the concatenated latent vector using a fully connected projection and four transposed-convolution blocks with a final tanh activation. The final latent dimensionalities were zc = 128 and zn = 64.

(3) LSVAE=Lrec+βc KL(qφ(Zc|X)||p(Zc))+βn KL(qφ(Zn|X)||pn(Zn))+λorth Lorth+λadv Ladv

To reduce leakage of diagnostic information into zn, an auxiliary adversarial classifier was trained on the nuisance head. The SVAE objective combined image reconstruction, KL divergence for both latent heads, an orthogonality penalty between zc and zn and the adversarial label-suppression term. The final loss weights were beta_KL = 1.0, lambda_orthogonality = 0.1 and lambda_adversarial = 0.5. Because complete disentanglement cannot be guaranteed from these losses alone, possible latent leakage is treated as a limitation and is not assumed away.

### 3.4. Heterogeneous Bayesian Experts

After disentanglement, three stochastic latent-space experts operate on complementary information streams. The causal expert receives zc and models lesion-relevant evidence. The nuisance-interaction expert receives zn and was implemented as a two-layer graph attention network [29]. Each image-level nuisance representation was treated as a node feature vector; edges were constructed using k-nearest-neighbor similarity in zn space with k = 8. Edge weights were computed from cosine similarity and normalized before message passing. Each graph-attention layer used four attention heads with hidden dimension 64, mean aggregation, dropout of 0.20 and a final classification head. The purpose of this expert was not to diagnose disease directly from artifacts, but to model nuisance-structure patterns that inform the Dirichlet fusion module when acquisition-related variation is high. The cross-covariate expert receives the concatenated latent representation [zc; zn] to capture residual interactions when acquisition conditions are relatively clean. 

Each expert is trained independently on frozen SVAE representations using cross-entropy loss under the same preprocessing, augmentation, data split and number-of-run protocol used for the baselines. Class imbalance was handled consistently across methods using stratified splits and class-balanced training weights computed from the training partition. For each test image, repeated stochastic forward passes generate a mean predictive vector and an epistemic-variability estimate, as summarized in Equations (4) and (5).

(4) $$\overset{-}{p}e(y|x)=(1/T)\Sigma t=1..T\;pe(t)(y|x)$$

(5) $$\mathrm{ue}=(1/T)\;\Sigma t=1..T\;(\mathrm{pe}(t)(y|x)-\overset{-}{p}e(y|x){)}^{2}$$

### 3.5. Dirichlet-Based Fusion with Backdoor Adjustment

The final component is a transformer-based meta-learner that aggregates expert predictions into a single Dirichlet distribution over class probabilities. Each expert contributes a feature vector containing its mean class prediction and stochastic variance. The fusion transformer used two encoder layers, a model dimension of 64, four attention heads and a feed-forward classification head. The output evidence was constrained to be non-negative using a softplus activation and converted into Dirichlet concentration parameters according to Equation (6).

αk = ek + 1, ek = softplus(hk)(6)

(7) $$\hat{p}k=\alpha k/\Sigma j\alpha j$$

**Figure 1:**
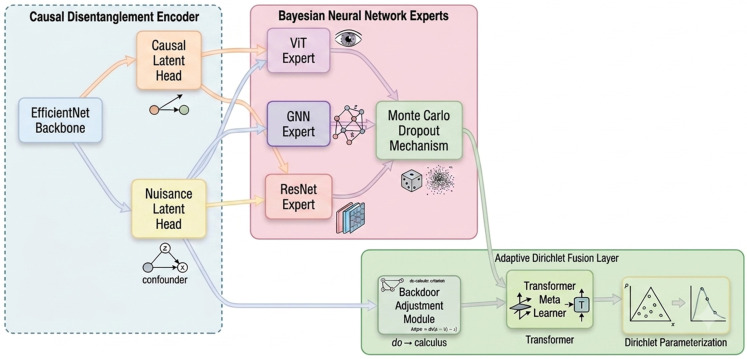
Internal architecture of the causal uncertainty-decomposed ensemble. The light-blue dashed region represents the causal disentanglement encoder, which generates lesion-relevant and acquisition-related nuisance latent representations. The pink region represents the three stochastic expert networks and the Monte Carlo dropout mechanism, whereas the light-green region represents the backdoor-adjustment module, transformer-based meta-learner and Dirichlet fusion stage. Peach/orange arrows indicate the flow of lesion-relevant latent information, while blue and yellow arrows indicate nuisance-related and cross-covariate information pathways. Purple and gray arrows represent the transfer of expert predictions and stochastic uncertainty estimates, and green arrows indicate the final uncertainty-aware fusion pathway. Arrowheads show the direction of information processing.

The backdoor-adjusted training objective exposes the fusion module to nuisance configurations sampled from the empirical reference distribution, using four nuisance samples per batch. This discourages dependence on specific artifact patterns and encourages the meta-learner to identify predictions that remain stable when nuisance features vary. The final uncertainty score is inversely related to total Dirichlet strength, as shown in Equation (8); high uncertainty can trigger optional deferral in selective-classification experiments. The complete CUDE architecture and the direction of information flow across its principal computational stages are summarized in Figure 1.

U = K/Σj αj(8)

### 3.6. Training Procedure

The CUDE framework was implemented in Python version 3.12.10 using PyTorch version 2.7.1, torchvision version 0.22.1 and PyTorch Geometric version 2.7.0. Statistical analyses were performed using scikit-learn version 1.7.2, while GPU-accelerated model training was conducted using CUDA version 12.6 and cuDNN version 9.6.0 on an NVIDIA A100 GPU. Training was completed in three modular stages. First, the SVAE was pre-trained on the dermoscopic image dataset for 200 epochs using the AdamW optimizer, a learning rate of 1.0 × 10^−4^, a weight decay of 1.0 × 10^−4^, a batch size of 32 and a cosine-annealing learning-rate schedule. Second, the three stochastic experts were trained independently on frozen latent representations for 100 epochs using AdamW with a learning rate of 3.0 × 10^−4^. Third, the Dirichlet fusion module was trained for 50 epochs using AdamW with a learning rate of 1.0 × 10^−4^. Unless otherwise specified, all comparative models were evaluated using the same image-resizing, normalization, augmentation, data-splitting, class-balancing and statistical-testing procedures. Five independent runs were conducted for the main comparative experiments. The selective-classification experiments were evaluated at uncertainty-based deferral thresholds of 10% and 20%, which were calibrated on the validation partition using Dirichlet strength. 

### 3.7. Dataset, Preprocessing and Experimental Setup

The framework was evaluated on the publicly available ISIC 2019 Challenge training collection, an established benchmark for dermoscopic image classification [30]. he labeled collection contains 25,331 dermoscopic images across eight diagnostic categories: melanoma (MEL), melanocytic nevus (NV), basal cell carcinoma (BCC), actinic keratosis (AK), benign keratosis (BKL), dermatofibroma (DF), vascular lesion (VASC) and squamous cell carcinoma (SCC). Because official hidden-test labels were not available for independent analysis, the experimental protocol used a stratified internal split of the labeled ISIC 2019 training data: 70% training, 10% validation and 20% held-out testing, with seed 42. The exact split-manifest format is provided in the accompanying repository. Images were resized to 256 × 256 pixels and normalized to the range [−1, 1]. Standard data augmentation included random horizontal flips, random rotations up to 30 degrees and color jitter.

The nuisance-reference set was constructed from the training partition only and was kept disjoint from the validation and held-out test partitions. It contained training images with visually apparent acquisition-related nuisance patterns, including terminal hairs, ruler or measurement markings, illumination variation, shadows and gel or reflection artifacts. This reference set was used only to estimate nuisance variation and to sample nuisance configurations during fusion training; it was not used as an additional labeled test set. The synthetic corruption protocol used in the robustness experiment was designed to approximate a subset of these nuisance patterns under controlled conditions, but it does not represent the full range of real-world dermoscopic acquisition shift.

### 3.8. Evaluation Metrics, Robustness Testing and Statistical Analysis

Diagnostic performance was assessed using balanced accuracy, AUROC, sensitivity and specificity. Calibration was evaluated using expected calibration error (ECE), Brier score and negative log-likelihood (NLL) [31]. Robustness was evaluated using a predefined synthetic corruption protocol applied to 500 stratified clean test images. The protocol added one of three acquisition-like artifacts: randomly drawn hair masks, simulated measurement scales placed near the lower image field and Gaussian-blurred light-reflection overlays. This protocol was intended as a controlled stress test rather than a complete model of real-world acquisition shift. Relative performance degradation, ECE, Brier score and NLL were computed using Equations (9)–(12). Statistical comparisons used paired t-tests with Bonferroni correction across repeated runs. For selective classification, the 10% and 20% deferral thresholds were based on the highest uncertainty scores, and sensitivity, specificity and false-negative rate were reported for melanoma, BCC and SCC in the non-deferred subset.

### 3.9. Computational Complexity and Reproducibility

Because CUDE uses multiple stochastic experts, model complexity was assessed using parameter count, mean inference time per image and peak GPU memory. Measurements were obtained on the same NVIDIA A100 GPU used for the benchmark experiments with batch size 1 for latency reporting. The inference-time path uses the SVAE encoder, three latent-space experts and the Dirichlet fusion module; the decoder is required for representation learning and latent inspection but is not required for routine classification after training. The main source of additional inference cost is the use of 30 Monte Carlo dropout passes per expert. Therefore, CUDE is more computationally demanding than a single deterministic classifier, and real-time clinical use would require batching, pass-number reduction, distillation or hardware-specific optimization. No claim of clinically real-time throughput is made in this study.

The public repository reports the same core configuration as the manuscript: stratified ISIC 2019 internal split, image size 256, zc = 128, zn = 64, dropout = 0.20, 30 Monte Carlo passes, beta_KL = 1.0, lambda_orthogonality = 0.1, lambda_adversarial = 0.5, two-layer transformer fusion with four attention heads, four nuisance samples per batch, five independent runs for comparative evaluation and 10% and 20% validation-calibrated deferral thresholds.

Relative drop (%) = [(BACclean − BACcorrupted)/BACclean] × 100(9)

ECE = Σm = 1..M (|Bm|/N) |acc(Bm) − conf(Bm)|(10)

Brier score = (1/N) Σi = 1..N Σk = 1..K (pik − yik)^2^ (11)

NLL = −(1/N) Σi = 1..N log p(i, yi)(12)

## 4. Results

The results are organized around four questions: whether CUDE improves benchmark classification performance on the held-out ISIC 2019 internal test partition, whether it reduces degradation under the predefined synthetic artifact protocol, whether the latent spaces show measurable separation and whether the Dirichlet fusion module produces calibrated uncertainty estimates suitable for exploratory selective classification.

### 4.1. Comparative Diagnostic Performance

As shown in Table 1, CUDE achieved the highest balanced accuracy (89.7 ± 0.4%) and AUROC (0.972 ± 0.002) among the compared methods. EfficientNet-B4 was the strongest single-model baseline, with a balanced accuracy of 85.1 ± 0.5%, whereas the majority-voting ensemble reached 86.4 ± 0.4%. The conventional deep ensemble of five ResNet-152 models achieved 87.3 ± 0.6%, indicating that standard ensembling improved performance but did not match the proposed structured approach under the same benchmark protocol.

The performance gain of CUDE over the baselines was statistically significant (*p* < 0.01 after Bonferroni correction). Class-level gains were most pronounced for melanoma and basal cell carcinoma, with sensitivities of 86.2% and 92.5%, respectively, compared with 81.4% and 89.1% for the deep ensemble. These results are consistent with the hypothesis that artifact-aware latent structuring may reduce reliance on occluding hairs and superficial dermoscopic artifacts; however, they should not be interpreted as proof of causal disentanglement.

### 4.2. Robustness to Confounding Artifacts

The corrupted test-set experiment assessed whether performance deteriorated when predefined acquisition-like artifacts were superimposed on clean dermoscopic images. As shown in Table 2, CUDE had a relative performance drop of 5.0%, which was smaller than the degradation observed for the deep ensemble (9.6%) and all single-model baselines. This finding supports artifact-stress robustness within the tested corruption protocol, but the claim is bounded by the limited artifact types and image subset used.

Internal expert analysis showed that the causal latent expert maintained 88.1% balanced accuracy under corruption, whereas the cross-covariate expert operating on concatenated features decreased to 79.3%. The fusion module increased the Dirichlet concentration assigned to the more stable expert when nuisance features were extreme, indicating adaptive expert reweighting under artifact stress.

**Figure 2:**
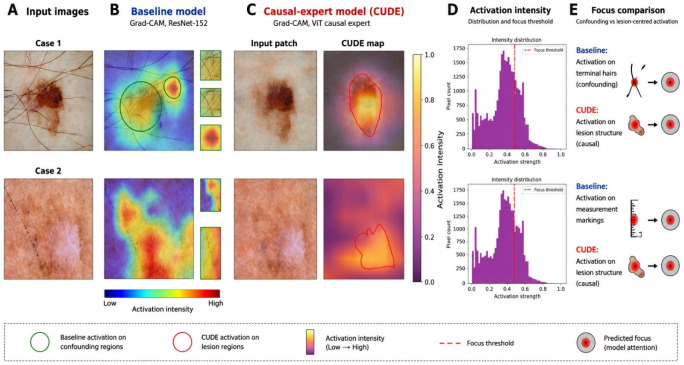
Activation-focus comparison between the baseline model and the causal-expert model on dermoscopic images containing terminal hairs and measurement markings.

Qualitative saliency analysis indicated that the baseline ResNet-152 model attended to nuisance regions such as hairs and ruler edges, whereas the causal expert focused more strongly on lesion borders and color variegation (Figure 2). This visual evidence is supportive but not definitive; it is interpreted together with the quantitative robustness and ablation results.

### 4.3. Uncertainty Quantification and Calibration

Calibration is important for selective classification because predicted probabilities should reflect the likelihood of correctness [31]. CUDE achieved the lowest ECE, Brier score and NLL among the compared methods (Table 3). The ECE of 0.039 represents a 33% and 47% reduction relative to the deep ensemble and Monte Carlo dropout ResNet-152 baseline, respectively.

**Figure 3:**
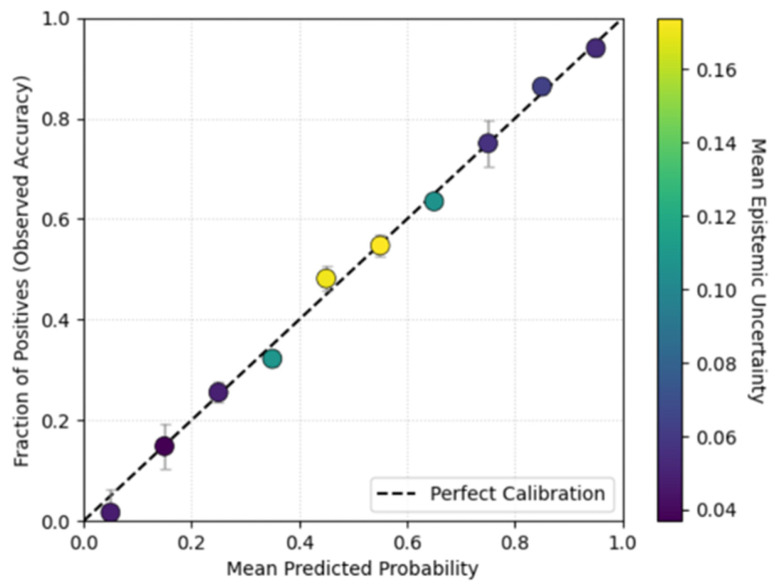
Reliability diagram for CUDE. Sample density is shaded according to epistemic uncertainty, and the diagonal line indicates perfect calibration.

The reliability diagram showed close agreement with the perfect-calibration diagonal across most confidence bins, with slight overestimation in the highest confidence interval (Figure 3). Deferral analysis showed that deferring the 10% most uncertain cases increased accuracy among non-deferred cases from 89.7% to 93.2%; at a 20% deferral rate, non-deferred accuracy increased to 95.1%. As shown in Table 4, malignant-class sensitivity increased and false-negative rates decreased for melanoma, BCC and SCC in the non-deferred subset at both deferral thresholds. High-confidence incorrect predictions remained present but were less frequent after deferral, indicating that the uncertainty score preferentially selected a portion of difficult or ambiguous cases. These thresholds are exploratory and would need local calibration to clinical workflow capacity, malignant-class sensitivity requirements and acceptable false-negative risk before clinical use.

### 4.4. Ablation Study

Ablation experiments examined the contribution of the Dirichlet fusion module, nuisance sampling, SVAE structure, auxiliary loss and expert ensemble. Table 5 shows that replacing the SVAE with a standard variational autoencoder produced the largest degradation, particularly under corrupted conditions. Removing nuisance sampling increased corruption sensitivity, and removing Dirichlet fusion degraded both robustness and calibration. The single-expert variant retained high corrupted-set balanced accuracy but had lower clean-set accuracy and poorer calibration than full CUDE, indicating that the complete framework provided the strongest overall benchmark trade-off. These ablations provide internal evidence that the proposed components contribute to performance, but they do not by themselves prove full semantic disentanglement of zc and zn.

### 4.5. Direct Latent-Separation Check

To provide a direct check of latent separation, simple diagnostic probes were trained on frozen latent representations. As shown in Table 6, the zc representation retained substantially higher diagnostic information than zn, whereas zn alone showed limited but non-zero classification performance. This pattern supports the intended separation of lesion-relevant and nuisance-dominant information, but it also indicates possible residual latent leakage. Therefore, the manuscript does not claim complete disentanglement.

### 4.6. Computational Complexity and Scope of Evidence

Table 7 summarizes the computational implications of the CUDE configuration evaluated in this study. The additional cost is driven primarily by repeated stochastic forward passes rather than by the latent-vector experts themselves. The uncertainty-aware CUDE configuration required 418.5 ± 18.7 ms per image on an NVIDIA A100 GPU, compared with 8.7 ± 0.5 ms for ResNet-152 and 43.6 ± 2.1 ms for the five-model ResNet-152 deep ensemble. This trade-off is acceptable for benchmark evaluation but must be optimized before any time-sensitive clinical workflow can be considered.

## 5. Discussion

The results suggest that CUDE improves dermoscopic classification within the evaluated ISIC 2019 internal test split by addressing a key limitation of conventional deep learning systems: reliance on artifact-driven shortcuts. The gain in balanced accuracy, smaller performance drop under the predefined synthetic corruption protocol, improved calibration, direct latent-probe results and improved malignant-class performance after uncertainty-based deferral collectively indicate that structured latent representation and uncertainty-aware fusion can improve benchmark robustness and selective classification. These findings should be interpreted as controlled experimental evidence, not as evidence of readiness for clinical screening across heterogeneous real-world settings.

### 5.1. Interpreting the Role of Causal Disentanglement

The ablation and latent-probe analyses indicate that the SVAE is central to the framework. When it was replaced with a vanilla variational autoencoder, clean-set accuracy declined and corrupted-set accuracy decreased more substantially, indicating that simple latent factorization is insufficient when nuisance and lesion-relevant signals are entangled. The zc-only diagnostic probe retained substantially more disease-relevant information than the zn-only probe, supporting the intended direction of latent separation. However, zn-only performance remained above chance, indicating residual diagnostic leakage. The results therefore demonstrate improved behavior under the imposed structure rather than formal proof that zc and zn are perfectly disentangled.

### 5.2. Expert Diversity and Robustness

The heterogeneous ensemble indicates that specialized stochastic experts can improve robustness when they provide complementary information. The causal latent expert functions as a stable anchor under artifact stress, whereas the cross-covariate expert contributes residual information when acquisition conditions are relatively clean. The nuisance-interaction expert helps the fusion module calibrate expectations about artifact configurations. The transformer fusion module then uses stochastic variance and Dirichlet evidence to adjust the influence of each expert dynamically instead of relying on static voting.

### 5.3. Clinical Relevance and Deployment Considerations

Several practical considerations remain before any clinical use. First, the deferral threshold must be defined in relation to dermatologist availability, patient triage urgency and the relative costs of false-negative and false-positive decisions, especially for melanoma, BCC and SCC. In the present benchmark, deferral improved malignant-class sensitivity and reduced false-negative rates in the non-deferred subset, but these thresholds are not clinical operating points. Second, the computational demands of Monte Carlo dropout across multiple experts were measurable: the final CUDE setting required substantially higher latency and memory than single-model baselines. Practical use would require model distillation, batching, fewer stochastic passes or hardware optimization. Third, the nuisance prior may not capture all artifact types encountered in real-world practice, including poor contact, motion blur, ink markings, device-specific color profiles, surgical sutures, tattoos or wound dressings. These considerations support the need for external multi-center validation and open-set artifact detection before the framework is used in clinical decision support.

### 5.4. Limitations

This study has several limitations. The evaluation was based on ISIC 2019 and a synthetically corrupted test subset rather than prospectively acquired multi-center clinical data. No external dataset, prospective cohort, explicit device-stratified test set or skin-tone-diverse validation was performed. ISIC 2019 is known to be dominated by lighter skin phototypes, and this study did not evaluate performance separately across Fitzpatrick IV-VI or across specific dermoscope/device families. The robustness experiment therefore approximates selected acquisition-related artifacts but does not fully represent real-world distribution shift, device variation, contact artifacts, motion blur, ink markings, tattoos, wound dressings or diagnostic-equity questions in darker phototypes. In addition, the interpretation of causal disentanglement depends on the validity of the assumed structural model and on the degree to which the latent subspaces successfully separate lesion-relevant features from nuisance artifacts. The direct probe analysis showed reduced but non-zero diagnostic information in zn, so latent leakage remains a genuine failure mode, especially when artifacts structurally overlap with lesion morphology, such as hairs overlying melanoma. Finally, although latency, parameter count and memory are reported, clinically realistic throughput was not prospectively benchmarked in a live workflow and requires future evaluation.

## 6. Conclusions

This study presents CUDE, a causal-prior and uncertainty-aware ensemble for skin-lesion classification in dermoscopic images. By encouraging separation of lesion-relevant features from nuisance artifacts, assigning stochastic experts to complementary latent streams and using a Dirichlet fusion module with nuisance sampling, the method improved accuracy, controlled-artifact robustness and calibration relative to the evaluated baselines on the ISIC 2019 internal test split. CUDE achieved 89.7% balanced accuracy, 0.972 AUROC, 5.0% relative performance degradation under the predefined synthetic artifact protocol and an ECE of 0.039. Deferring the 10% most uncertain cases increased non-deferred accuracy to 93.2% and reduced malignant-class false-negative rates in the non-deferred subset. These results support further investigation of structured representation learning and uncertainty-aware selective classification, but they do not establish broad real-world robustness, complete causal disentanglement or clinical deployment readiness. External multi-center validation, prospective evaluation under real acquisition conditions, skin-tone-diverse testing, device-stratified analysis and computational optimization are required before translation into clinical decision support.

## Figures and Tables

**Table 1:** Classification performance on the held-out ISIC 2019 internal test partition. Values are reported as mean ± standard deviation over five independent runs.

Method	Balanced Accuracy (%)	AUROC	Sensitivity	Specificity
ResNet-152 [3]	84.3 ± 0.6	0.954 ± 0.004	0.812	0.974
EfficientNet-B4 [32]	85.1 ± 0.5	0.959 ± 0.003	0.823	0.976
DenseNet-121 [33]	84.7 ± 0.7	0.957 ± 0.005	0.819	0.975
Majority Voting Ensemble [13]	86.4 ± 0.4	0.964 ± 0.003	0.841	0.978
Stacking Ensemble [14]	86.1 ± 0.5	0.962 ± 0.004	0.837	0.977
Deep Ensemble (ResNet-152 × 5) [12]	87.3 ± 0.6	0.967 ± 0.003	0.855	0.979
FactorVAE Disentanglement [20]	83.9 ± 0.8	0.948 ± 0.006	0.807	0.971
CUDE (Proposed)	89.7 ± 0.4	0.972 ± 0.002	0.881	0.982

**Table 2:** Robustness to the predefined synthetic artifact protocol. Relative drop was computed using Equation (9).

Method	Corrupted BAC (%)	Relative Drop (%)
ResNet-152	73.1 ± 1.2	13.3
EfficientNet-B4	74.8 ± 0.9	12.1
Majority Voting Ensemble	76.5 ± 1.0	11.5
Deep Ensemble (ResNet-152 × 5)	78.9 ± 0.8	9.6
FactorVAE Disentanglement	72.3 ± 1.4	13.8
CUDE (Proposed)	85.2 ± 0.7	5.0

**Table 3:** Calibration and uncertainty metrics on the held-out ISIC 2019 internal test partition. Lower values indicate better calibration or sharper probabilistic prediction. The downward arrow (↓) denotes that lower values are preferable.

Method	ECE ↓	Brier Score ↓	NLL ↓
ResNet-152 (MC-dropout)	0.074 ± 0.008	0.231	0.742
Deep Ensemble (ResNet-152 × 5)	0.058 ± 0.006	0.214	0.694
FactorVAE Disentanglement	0.089 ± 0.011	0.249	0.801
CUDE (Proposed)	0.039 ± 0.005	0.189	0.613

**Table 4:** Malignant-class performance after uncertainty-based deferral for CUDE on the held-out ISIC 2019 internal test partition. FNR, false-negative rate.

Class	No Deferral: Sensitivity/Specificity/FNR (%)	10% Deferral: Sensitivity/Specificity/FNR (%)	20% Deferral: Sensitivity/Specificity/FNR (%)
Melanoma	86.2/98.1/13.8	90.8/98.6/9.2	93.5/99.0/6.5
BCC	92.5/98.4/7.5	95.1/98.8/4.9	96.8/99.1/3.2
SCC	85.7/97.9/14.3	89.4/98.4/10.6	92.1/98.8/7.9

**Table 5:** Ablation study analyzing the contribution of each component. Values are reported as mean ± standard deviation over three runs.

Variant	Clean BAC (%)	Corrupted BAC (%)	ECE
Full CUDE	89.7 ± 0.4	85.2 ± 0.7	0.039 ± 0.005
No Dirichlet Fusion	87.9 ± 0.6	82.4 ± 0.9	0.062 ± 0.008
No Backdoor Adjustment	88.5 ± 0.5	80.1 ± 1.1	0.051 ± 0.006
Vanilla VAE	86.2 ± 0.7	77.8 ± 1.3	0.073 ± 0.009
No Auxiliary Loss	88.1 ± 0.5	83.6 ± 0.8	0.045 ± 0.006
Single Expert (ViT on Zc)	87.3 ± 0.5	86.1 ± 0.5	0.058 ± 0.007

**Table 6:** Diagnostic information retained in the disentangled latent spaces. Values are reported over repeated runs and are used as a direct probe of latent separation rather than as a deployment model.

Representation Used	Classifier/Probe	Balanced Accuracy (%)	AUROC	Interpretation
zc only	Linear/MLP diagnostic probe	86.9 ± 0.6	0.958 ± 0.004	Strong disease-relevant information retained
zn only	Linear/MLP diagnostic probe	37.4 ± 1.3	0.681 ± 0.011	Limited but non-zero diagnostic leakage
[zc; zn]	Linear/MLP diagnostic probe	88.1 ± 0.5	0.964 ± 0.003	Complementary information retained
Full CUDE	Complete fusion model	89.7 ± 0.4	0.972 ± 0.002	Best overall performance

**Table 7:** Computational complexity and practical inference characteristics for CUDE and selected baselines.

Model	Parameters (M)	MC Passes	Mean Inference Time per Image (ms)	Peak GPU Memory (GB)
ResNet-152	58.1	1	8.7 ± 0.5	2.3
EfficientNet-B4	19.3	1	7.9 ± 0.4	2.1
Deep ensemble (ResNet-152 × 5)	290.5	1 per model	43.6 ± 2.1	6.4
CUDE, deterministic	164.8	1	21.4 ± 1.3	4.8
CUDE, MC dropout	164.8	30	418.5 ± 18.7	7.9

## Data Availability

The original ISIC 2019 image data are publicly available from the ISIC Archive and are not redistributed with this manuscript. The accompanying repository provides the dataset-preparation script, split-manifest format, configuration file, model code and synthetic-corruption protocol used to reproduce the reported workflow after users download the public ISIC 2019 data from the official source. The repository is available at: https://github.com/drfaheem-007/CUDE_Git_Package (accessed on 10 June 2026).

## Abbreviations

The following abbreviations are used in this manuscript:

AUROC, area under the receiver operating characteristic curve; BAC, balanced accuracy; BCC, basal cell carcinoma; BKL, benign keratosis; CUDE, causal uncertainty-decomposed ensemble; DF, dermatofibroma; ECE, expected calibration error; FNR, false-negative rate; GPU, graphics processing unit; GNN, graph neural network; ISIC, International Skin Imaging Collaboration; MEL, melanoma; MC dropout, Monte Carlo dropout; NLL, negative log-likelihood; NV, melanocytic nevus; SCC, squamous cell carcinoma; SVAE, structured variational autoencoder; VAE, variational autoencoder; VASC, vascular lesion; ViT, Vision Transformer.
